# Behavioral Inhibition and Social Anxiety Disorder as Predictors of Long-Term Outcomes of Cognitive Behavioral Therapy for Youth Anxiety Disorders

**DOI:** 10.1007/s10802-024-01215-8

**Published:** 2024-06-05

**Authors:** Toril Skumsnes, Krister W. Fjermestad, Gro Janne Wergeland, Marianne Aalberg, Einar R. Heiervang, Arne Kodal, Jo Magne Ingul

**Affiliations:** 1 Tynset Child and Adolescents Mental Health Service, Innlandet Hospital Trust, Tynset, Norway; 2 Department of Mental Health, Norwegian University of Science and Technology, Trondheim, Norway; 3 Department of Psychology, University of Oslo, Oslo, Norway; 4 Department of Child and Adolescent Psychiatry, Division of Psychiatry, Haukeland University Hospital, Bergen, Norway; 5 Department of Clinical Medicine, Faculty of Medicine, University of Bergen, Bergen, Norway; 6 Department for Research and Development, Division of Mental Health and Substance Use, Akershus University Hospital, Oslo, Norway; 7 Regional Centre for Child and Youth Mental Health and Child Welfare, NORCE Norwegian Research Centre, Bergen, Norway

**Keywords:** Behavioral Inhibition, Social Anxiety Disorder, Anxiety Disorders, CBT, Youth

## Abstract

The temperamental trait behavioral inhibition (BI) is related to the development and maintenance of anxiety, particularly much so to social anxiety disorder. We investigated if BI and social anxiety disorder predicted cognitive behavioral therapy (CBT) outcomes for youth anxiety. Youth (*N* = 179; M_age_ = 11.6 years) were assessed 4 years following a randomized controlled CBT effectiveness trial. BI was measured by the parent-reported Behavioral Inhibition Questionnaire at baseline. The outcomes were diagnostic recovery, youth- and parent-reported anxiety symptoms, and clinical severity at post-treatment, 1-year, and 4-year follow-up. Having social anxiety disorder negatively predicted diagnostic recovery and predicted higher clinical severity at all assessment points and was the only significant predictor of outcomes at 4-year follow-up. Higher BI negatively predicted diagnostic recovery and predicted higher clinical severity and parent-reported symptom levels at post-treatment and 1-year follow-up, and predicted higher youth-reported anxiety levels at 1-year follow-up. Higher BI was the only predictor of youth- and parent-reported anxiety symptoms. BI and social anxiety disorder seem to be unique predictors of CBT outcomes among youth with anxiety disorders. CBT adaptations may be indicated for youth with high BI and social anxiety disorder.

Cognitive behavioral therapy (CBT) for youth anxiety is well-established with a strong evidence base (Higa-McMillan et al., 2016). However, CBT is insufficient for a substantial group of youth with anxiety disorders (Baker et al., 2021; Heiervang et al., 2018; Wergeland et al., 2021). In a study of long-term outcomes of CBT treatment in youth, only 22% achieved stable recovery in terms of the absence of any DSM-IV-TR diagnoses over 4 years 4–16 years after treatment (Ginsburg et al., 2018). Importantly, clinical improvement of anxiety symptoms post-treatment reduced the probability of later chronic anxiety in terms of having an anxiety disorder at every assessment point during the 4 years (Ginsburg et al., 2018). This finding highlights the pervasive nature of anxiety disorders and the need to improve anxiety treatments, which has been echoed by recent calls for more research (Baker et al., 2021; Wergeland et al., 2021). Disentangling factors that negatively influence treatment outcomes may be an approach toward better understanding treatment effects and improving treatment outcomes for youth.

Among factors that predict treatment outcomes, social anxiety has been identified as a significant predictor of poor treatment outcomes across numerous studies (Compton et al., 2014; Evans et al., 2021; Hudson et al., 2015). Central to the development and maintenance of anxiety disorders in general and social anxiety disorder in particular is the temperamental predisposition behavioral inhibition (BI) (Sandstrom et al., 2020; Spence & Rapee, 2016), which is also a suggested predictor of treatment outcome for preschool children with anxiety (Hirshfeld-Becker et al., 2010). The two constructs social anxiety disorder and BI are theoretically and empirically related, but the nature of their relationship is uncertain (Fox et al., 2023; Pérez-Edgar & Guyer, 2014). Their shared and distinct features may inform our understanding of processes that hinder treatment effects and how to improve treatment.

Social anxiety disorder is characterized by a persisting and strong fear of social situations or performance settings where evaluation from others may occur and cause personal distress or interference with one’s everyday functioning (APA, 1994). A lifetime prevalence of 12.1% has been estimated, with 13 years as the median age of onset (Kessler et al., 2005). Social anxiety is associated with a potential chronic course, and with functional impairment within education, work, and/or social life (Aderka et al., 2012; Bruce et al., 2005). In a recent review and meta-analysis, recovery from youth social anxiety disorder after generic CBT was found to be significantly less likely compared to other anxiety disorders, with recovery rates of 35% compared to 54% for other anxiety disorders (Evans et al., 2021).

According to Wong and Rapee’s (2016) integrated etiology and maintenance (IAM) model of social anxiety disorder, an individual’s social functioning will be affected by the degree of threat that they assign to social situations. The model states that an individual’s threat assignment is determined by several etiological factors, of which temperament is proposed to have the greatest potential to influence this threat evaluation from birth to childhood. Further, an increase in the perception of danger attributed to social situations will encourage the development of self-focus, attentiveness to threats in the environment, avoidance of threatening situations, and the use of safety strategies (Wong & Rapee, 2016).

CBT for anxiety disorders in children and youth is often provided in a generic format including psychoeducation, anxiety management, cognitive restructuring, and graded exposure to feared situations (Arch & Craske, 2008). A traditional habituation-based model of exposure therapy proposes fear reduction during exposure trials as essential to changing the expectations of a feared situation. For youth with social anxiety disorder, there is a risk that techniques to reduce anxiety would serve as safety behaviors that reduce anxiety in the situation but at the same time maintain the disorder by establishing dependency on safety strategies to avoid the feared scenario (Craske et al., 2014).

BI is a temperamental predisposition limiting an individual’s behavior by a particular attentiveness to potential threats and heightened physiological reactivity in unfamiliar situations (APA, 2023). BI is an innate, and stable trait observable from early childhood which influences a person’s pattern of actions. An estimated 15–20% of infants are born with this trait (Kagan & Snidman, 1999). Children with high BI have a threefold increase in odds of developing anxiety disorders compared to other children (OR = 2.80, 95% CI 2.03, 3.86) (Sandstrom et al., 2020). The odds of developing a social anxiety disorder are even higher, close to a six-fold increase in odds compared to children without BI (OR = 5.84, 95% CI 3.38, 10.09) (Sandstrom et al., 2020). While BI predisposes an individual towards anxiety disorders, less than half of all infants with high BI later develop anxiety disorders, and not all with anxiety disorders have high BI (Muris et al., 2011).

BI is strongly implicated as a developmental factor in theoretical models of social anxiety disorder (Kimbrel, 2008; Ollendick & Benoit, 2012; Wong & Rapee, 2016). Certain behaviors that are central to BI are also seen as core traits of social anxiety disorder, such as behavioral withdrawal and avoidance, lack of eye contact and verbal utterances, and keeping proximity to attachment figures (Spence & Rapee, 2016). The tendencies to be more attentive to threats and to own errors are factors suggested to strengthen the relation of childhood BI to youth anxiety disorders and adult internalizing problems, and are also maintaining factors of social anxiety disorder (Fox et al., 2023; Warnock-Parkes et al., 2022; Wong & Rapee, 2016). The characteristic features of BI could be expected to affect the therapeutic process in terms of reactivity to, and withdrawal, from both interaction with the therapist and treatment tasks. Thus, a relevant question is whether BI is a negative predictor of treatment outcomes.

BI has been found to predict poorer outcomes from CBT among preschool children with heightened anxiety levels (Morgan et al., 2018; Hirshfeld-Becker et al., 2010). Extant literature on BI as a predictor of CBT outcomes among youth anxiety is scarce. Capriola and colleagues (2017) investigated temperamental profiles and outcomes from one-session CBT among youth with specific phobias. They found that improvement was comparable across temperamental profiles when comorbidity was accounted for (Capriola et al., 2017). Other studies involving BI-related traits, such as negative affect and shyness, did not find a predictive relation to CBT outcomes (Carper, 2020; Festen et al., 2013).

The close resemblance between BI and social anxiety disorder necessitates an awareness of social anxiety disorder being a confounding factor to the predictive relationship between BI and CBT outcomes for youth with anxiety disorders. To the best of our knowledge, no study has specifically investigated BI and social anxiety disorder as separate predictors of treatment outcome. At present, it is unclear whether having social anxiety in the diagnostic profile and BI are merely overlapping predictors, or if they each have a unique contribution to CBT outcomes in youth with anxiety disorders.

In the current study, we examine social anxiety disorder and BI as potential factors that explain the variance in outcomes for youth with anxiety disorders participating in a randomized controlled trial of individual and group CBT. The original study showed diagnostic recovery of 23% post-treatment, 37% at 1-year follow-up, and 53% at 4-year follow-up, and social anxiety disorder was a negative predictor of outcomes (Kodal et al., 2018; Wergeland et al., 2014). Herein, we investigate if BI, and the presence of social anxiety disorder in the diagnostic profile at baseline, both are uniquely associated with treatment outcomes above and beyond consideration of the other. Our first research question is: (1) Do BI and social anxiety disorder both contribute uniquely to predict parent- and youth-reported anxiety symptoms and clinical severity ratings (CSR) at post-treatment, 1-year, and 4-year follow-up? We hypothesize that both BI and social anxiety disorder will be associated with higher symptom levels and clinical severity ratings at all measurement points. Our second research question is: (2) Do BI and social anxiety disorder both contribute uniquely to predict diagnostic recovery at post-treatment and at 1- and 4-year follow-up? We hypothesize that BI and social anxiety disorder negatively predict diagnostic recovery.

## Methods

### Participants

The present study included 179 youth (52.5% girls, 47.5% boys) with anxiety disorders participating in a randomized controlled trial (RCT) comparing the effectiveness of individual CBT and group CBT versus waitlist in routine clinical care, of which 86% (*n* = 154) and 82% (*n* = 146) took part in the clinical assessments immediate and 1-year post-treatment and 89% (*n* = 159) participated in the clinical assessment in a long-term follow-up study 4 years after treatment (Kodal et al., 2018; Wergeland et al., 2014). The inclusion criteria in the RCT were age 8–15 years and a principal diagnosis according to DSM-IV criteria of social anxiety disorder, separation anxiety disorder, or generalized anxiety disorder. The exclusion criteria were pervasive developmental disorder, psychotic disorder, and/or intellectual disability. There were no significant differences in outcomes for diagnostic recovery or anxiety symptoms by treatment modality neither post-treatment nor at 1-year or 4-year follow-up (Kodal et al., 2018; Wergeland et al., 2014).

Informed written consent, including consent to be contacted for long-term follow-up, were obtained from all parents at the time of inclusion in the original RCT. At the same time assent from youth above age 12 years were obtained. Parents and youth were both given a compensation gift card at 4-year follow-up (USD$50). The study was approved by the Regional Committee for Medical and Health Ethics of Western Norway. Further descriptions of the RCT, including sample, procedure, methods, and outcome details are available elsewhere (Kodal et al., 2018; Wergeland et al., 2014).

### Measures

#### Diagnostic Interview

The ADIS-C/P (Silverman & Albano, 1996) modules on separation-, social- and generalized anxiety were used to assess inclusion diagnoses for participants aged < 18 years. Participants and parents were interviewed independently. The ADIS for DSM-IV (ADIS -IV-L) (Brown et al., 1994) was used for participants aged 18 years or above at 4-year follow-up. The clinical severity ratings (CSR; 0–8) for participants aged < 18 were assigned based on the combined parent and youth report, according to the manual. A random selection of 20% of the diagnostic interviews across all assessment points were re-rated by experts to whom the original assessors´ ratings were masked. The overall inter-rater agreement on the diagnostic interviews at all assessment points were very good, with kappa values ranging from 0.83 to 0.94 (Kodal et al., 2018; Wergeland et al., 2014).

#### Spence Children’s Anxiety Scale; Child and Parent Version

The Spence Children’s Anxiety Scale; child and parent versions (SCAS-C/P) (Nauta et al., 2004; Spence, 1998) was used to measure youth anxiety symptoms. The questionnaire consists of 38 items, rated on a 4-point scale (0 = never, 3 = always) with a maximum score of 114. In the current sample internal consistency was good, with alpha values ranging from  0.85 to 0.92 (parent) and  0.89 to 0.91 (youth) (Kodal et al., 2018; Wergeland et al., 2014).

#### Behavioral Inhibition Questionnaire

The Behavioral Inhibition Questionnaire (BIQ) (Bishop et al., 2003) was used to obtain retrospective parent ratings of BI. Parents were asked to rate their youth at preschool age (3–5 years). The BIQ consists of 30 items rated on a 6-point scale (1 = hardly ever, 6 = almost always), with a maximum score of 180. The total scores were used in the analyses (primarily the mother’s score (80%), the father’s score was inserted if the mother’s score was missing). Internal consistency in the current sample was α = 0.96 (mother) and α = 0.95 (father).

The BIQ was used at baseline, while the ADIS and SCAS were used at four assessment points: baseline, post-treatment, and 1- and 4-year follow-up.

### Treatment, Setting, Therapists and Assessors

The CBT program *FRIENDS for Life*, 4th edition was used in the study (Barrett, 2005). Participants were given either the child (aged 8–12) or the adolescent (aged 12–15) version, either as 10 weekly 60-minute individual sessions (91 participants) or 90-minute group sessions (88 participants).

The RCT was conducted at seven community child and adolescent mental health outpatient clinics in Norway. Therapy was provided by 17 therapists, of which only five had previously completed a formal 2-year CBT training. In the study, all therapists received training in terms of a two-day workshop on CBT and childhood anxiety disorders, and a two-day workshop on FRIENDS. The therapists treated two pilot cases approved by the supervisors before the study started and received supervision by licensed FRIENDS-therapists during the treatment phase of the study. Based on ratings of 20% of the videotaped therapy sessions, all therapists were found to have good adherence to and competence in the treatment protocol (Kodal et al., 2018; Wergeland et al., 2014). Assessment for inclusion and outcome was conducted by 16 clinicians at the clinics after training by certified interviewers.

### Analysis

Two separate sets of main analyses were conducted in R version 4.3.2. We ran nine hierarchical linear regression models predicting youth and parent-reported youth anxiety symptoms and clinical severity ratings at the three assessment points: post-treatment, 1-year, and 4-year follow-up. The levels of missing data across assessment points were 12% (BI), 11–18% (CSR), 19–24% (SCAS-C), and 18–26% (SCAS-P). For the continuous outcomes, we used full information maximum likelihood to estimate model parameters when some data is missing, using Lavaan (v 0.6.16; Rosseel, 2012). We conducted three hierarchical logistic regression models predicting diagnostic recovery at the same assessment points, across which the level of missing data ranged from 11 to 18%. For the logistic regression models, we used multivariate imputations via chained equations to impute missing values of BI, using the mice package (v3.16.0; van Buuren & Groothuis-Oudshoorn, 2011). The hierarchical regression models for each assessment point and outcome measure were set up in the following order: Model (1) age and gender; Model (2) age, gender and BI; Model (3) age, gender and social anxiety disorder anywhere in the diagnostic profile at baseline (i.e., as the primary, secondary, or tertiary anxiety disorder); and Model (4) age, gender, BI and social anxiety disorder. In a fifth model BI and social anxiety disorder were entered reversely (age, gender, social anxiety disorder and BI). The fifth model was not included in the tables as the results were the same as in Model 4.

## Results

Among the 179 participants, 130 had social anxiety disorder anywhere in their diagnostic profile at baseline, with a mean BIQ score of 126.23 and full diagnostic recovery rates of 21.43% post-treatment, 36.54% at 1-year follow-up, and 42.74% at 4-year follow-up. The mean BIQ score for the 49 participants with no social anxiety disorder in the profile was 107.85 and this group had full diagnostic recovery rates of 40.48% at post-treatment, 66.67% at 1-year follow-up, and 73.81% at 4-year follow-up.

Social anxiety disorder was the primary diagnosis for 83 participants (mean BIQ score = 131.99), separation anxiety disorder was the primary diagnosis for 59 participants (mean BIQ score = 117.61), and generalized anxiety disorder was the primary diagnosis for 37 participants (mean BIQ score = 103.16). Symptom measures and diagnostic recovery rates across diagnostic groups and assessment points are shown in Table 1.

**Table 1:** Symptom Measures and Diagnostic Recovery Rates across Diagnostic Groups and Assessment Points

Variables		Total sample *N* = 179	Any SAD^a^ *n* = 130	No SAD^b^ *n* = 49	Prim. SAD^c^ *n* = 83	Prim. SEP^d^ *n* = 59	Prim. GAD^e^ *n* = 37
		(mean, sd)	(mean, sd)	(mean, sd)	(mean, sd)	(mean, sd)	(mean, sd)
	Baseline						
BI		121.08, 36.83	126.23, 36.61	107.85, 34.38	131.99, 34.09	117.61, 37.07	103.16, 35.00
Youth-Reported Symptoms		36.31, 16.58	37.48, 16.77	33.26, 15.85	34.57, 15.50	38.12, 17.57	37.22, 17.36
Parent-Reported Symptoms		34.89, 12.63	35.37, 12.94	33.71, 11.87	33.84, 13.41	37.10, 12.83	33.59, 10.34
Clinical Severity Rating		6.97, 1.14	7.09, 1.08	6.63, 1.24	7.05, 1.16	6.97, 1.07	6.78, 1.23
	Post-Treatment						
Youth-Reported Symptoms		27.39, 15.32	28.30, 15.76	24.95, 13.96	26.45, 14.71	29.51, 17.37	25.59, 12.33
Parent-Reported Symptoms		27.34, 13.33	27.75, 13.53	26.31, 12.90	25.68, 11.98	29.59, 16.22	26.55, 8.56
Clinical Severity Rating		4.73, 2.41	5.05, 2.18	3.83, 2.81	5.26, 2.00	4.82, 2.59	3.28, 2.45
Diagnostic Recovery Percentage^f^		26.62%	21.43%	40.48%	26.09%	19.64%	41.38%
	1-Year Follow-Up						
Youth-Reported Symptoms		23.61, 17.55	25.07, 19.04	19.70, 12.08	22.38, 17.89	25.73, 19.64	22.08, 10.24
Parent-Reported Symptoms		23.12, 13.28	23.44, 14.09	22.33, 11.14	21.98, 13.55	25.42, 14.17	20.99, 9.86
Clinical Severity Rating		3.71, 2.59	4.25, 2.44	2.38, 2.51	4.31, 2.45	3.53, 2.81	2.67, 2.13
Variables		Total sample *N* = 179	Any SAD^a^ *n* = 130	No SAD^b^ *n* = 49	Prim. SAD^c^ *n* = 83	Prim. SEP^d^ *n* = 59	Prim. GAD^e^ *n* = 37
		(mean, sd)	(mean, sd)	(mean, sd)	(mean, sd)	(mean, sd)	(mean, sd)
Diagnostic Recovery Percentage^f^		45.21%	36.54%	66.67%	41.54%	41.82%	61.54%
	4-Year Follow-Up						
Youth-Reported Symptoms		24.53, 16.38	25.53, 16.69	21.87, 15.43	25.32, 17.66	24.78, 14.94	22.47,16.22
Parent-Reported Symptoms		21.59, 13.19	23.03, 13.17	18.13, 12.77	22.42, 13.90	21.98, 14.25	19.11, 9.27
Clinical Severity Rating		2.33, 3.13	2.85, 3.19	0.88, 2.44	3.36, 3.25	1.67, 2.89	1.15, 2.55
Diagnostic Recovery Percentage^f^		50.94%	42.74%	73.81%	45.21%	53.85%	58.82%

Note. This table demonstrates mean scores and standard deviances of BIQ (BI), SCAS-C, SCAS-P (youth- and parent-reported youth anxiety symptoms) and CSR (clinical severity ratings) at all assessment points and the percentage of full diagnostic recovery (recovery from all inclusion diagnoses) at post-treatment, 1-year follow-up and 4-year follow-up across different diagnostic groups

^a^ Participants with social anxiety disorder anywhere in profile (*n* = 130); ^b^ Participants without social anxiety in the diagnostic profile (*n* = 49); ^c^ Participants with a primary diagnosis of social anxiety disorder (*n* = 83); ^d^ Participants with a primary diagnosis of separation anxiety disorder (*n* = 59); ^e^ Participants with a primary diagnosis of generalized anxiety disorder (*n* = 37); ^f^ Percentage of sample recovered from all inclusion diagnoses

BI total scores were not significantly different for those receiving individual CBT and group CBT. Furthermore, the treatment cluster variance for BI was ICC = 0.19, which is not so high as to suggest influential nesting effects (Guo, 2005).

### Predictors of Anxiety Symptoms and Clinical Severity

The results of the models with youth-reported youth anxiety symptoms as outcomes are shown in Table 2.

**Table 2:** Regression Models Predicting Youth-Reported Youth Anxiety Symptoms at Immediate, 1-Year, and 4-Year Post-Treatment

			Model 1		Model 2		Model 3		Model 4^a^	
			B (SE)	p	B (SE)	p	B (SE)	p	B (SE)	p
			Post-Treatment (*N* = 179)							
Predictors										
	Intercept		24.63 (7.12)	0.001**	25.20 (7.89)	0.001**	24.75 (7.09)	0.000***	26.12 (7.90)	0.001**
	Age		-0.05 (0.61)	0.939	-0.03 (0.62)	0.962	-0.26 (0.64)	0.689	-0.23 (0.64)	0.717
	Gender		6.34 (2.51)	0.011*	6.32 (2.51)	0.012*	6.24 (2.50)	0.013*	6.18 (2.50)	0.014*
	BI				-0.06 (0.36)	0.865			-0.14 (0.37)	0.694
	Social Anxiety Disorder						0.80 (0.73)	0.275	0.86 (0.75)	0.250
Model fit										
	AIC		2228.78		3089.53		2931.17		3788.07	
	R^2^		0.04		0.04		0.05		0.05	
			1-Year Follow-Up (*N* = 179)							
Predictors										
	Intercept		1.54 (0.83)	0.065	0.64 (0.89)	0.472	1.53 (0.83)	0.065	0.69 (0.89)	0.435
	Age		0.04 (0.07)	0.583	0.01 (0.07)	0.921	0.01 (0.07)	0.900	-0.01 (0.07)	0.884
	Gender		0.72 (0.29)	0.014*	0.75 (0.29)	0.010*	0.71 (0.29)	0.015*	0.74 (0.29)	0.010*
	BI				0.10 (0.04)	0.013*			0.09 (0.04)	0.023*
	Social Anxiety Disorder						0.12 (0.09)	0.151	0.08 (0.09)	0.327
Model fit										
	AIC		1572.99		2427.78		2274.53		3126.54	
	R^2^		0.05		0.09		0.06		0.10	
			4-Year Follow-Up (*N* = 179)							
Predictors										
	Intercept		0.94 (0.71)	0.182	1.29 (0.79)	0.106	0.96 (0.71)	0.174	1.34 (0.81)	0.095
	Age		0.09 (0.06)	0.141	0.10 (0.06)	0.103	0.08 (0.07)	0.230	0.09 (0.07)	0.190
	Gender		0.87 (0.27)	0.001**	0.85 (0.27)	0.001**	0.87 (0.27)	0.001**	0.85 (0.27)	0.001**
	BI				-0.04 (0.04)	0.348			-0.04 (0.04)	0.323
	Social Anxiety Disorder						0.04 (0.08)	0.589	0.06 (0.08)	0.485
Model fit										
	AIC		1569.24		2429.16		2272.53		3128.61	
	R^2^		0.09		0.10		0.09		0.10	

Note.^a^ In a fifth model, in which BI and Social Anxiety Disordered were entered reversely with Social Anxiety Disorder coming first, results were the same as in Model 4

*The predictor is significant at the *p* <.05 level. ** The predictor is significant at the *p* <.01 level. *** The predictor is significant at the *p* <.001 level

For youth-reported youth anxiety symptoms, gender was a significant predictor of symptom levels at all three assessment points, with higher symptom levels for girls. Model 1 (age and gender) seems to have the best fit for predicting post-treatment (AIC = 2228.78, explained variance (*r*^*2*^) = 4.3%) and 4-year follow-up outcomes (AIC = 1569.24, explained variance (*r*^*2*^) = 9.6%). BI was a significant predictor of outcomes at 1-year follow-up, with higher symptom levels for those with higher BI scores. Model 2 (age, gender, and BI) seems to have the best fit (AIC = 2427.78, explained variance (*r*^*2*^) = 9.4%).

The results of the models with parent-reported youth anxiety symptoms as outcome are shown in Table 3.

**Table 3:** Regression Models Predicting Parent-Reported Youth Anxiety Symptoms at Immediate, 1-Year, and 4-Year Post-Treatment

			Model 1		Model 2		Model 3		Model 4^a^	
			B (SE)	p	B (SE)	p	B (SE)	p	B (SE)	p
			Post-Treatment (*N* = 179)							
Predictors										
	Intercept		27.91 (6.19)	0.000***	18.12 (6.50)	0.005**	27.98 (6.19)	0.000***	18.01 (6.54)	0.006**
	Age		-0.03 (0.54)	0.951	-0.37 (0.52)	0.482	-0.14 (0.56)	0.800	-0.36 (0.54)	0.510
	Gender		-0.37 (2.21)	0.866	-0.13 (2.13)	0.952	-0.42 (2.21)	0.851	-0.13 (2.13)	0.950
	BI				1.09 (0.31)	0.000***			1.09 (0.31)	0.000***
	Social Anxiety Disorder						0.42 (0.64)	0.516	-0.03 (0.63)	0.963
Model fit										
	AIC		2210.98		3059.87		2914.14		3759.56	
	R^2^		0.00		0.09		0.00		0.09	
			1-Year Follow-Up (*N* = 179)							
Predictors										
	Intercept		21.87 (6.46)	0.001**	14.31 (6.81)	0.036*	21.86 (6.46)	0.001*	14.08 (6.84)	0.040*
	Age		0.02 (0.55)	0.974	-0.27 (0.55)	0.621	-0.05 (0.57)	0.935	-0.24 (0.56)	0.672
	Gender		1.95 (2.25)	0.387	2.01 (2.19)	0.358	1.92 (2.25)	0.394	2.04 (2.19)	0.351
	BI				0.88 (0.32)	0.006**			0.89 (0.32)	0.006**
	Social Anxiety Disorder						0.27 (0.64)	0.674	-0.11 (0.64)	0.859
Model fit										
	AIC		2153.21		3006.71		2856.61		3706.42	
	R^2^		0.01		0.06		0.01		0.06	
			4-Year Follow-Up (*N* = 179)							
Predictors										
	Intercept		18.61 (6.46)	0.004**	15.51 (7.11)	0.029*	19.37 (6.38)	0.002**	16.87 (7.10)	0.018*
	Age		0.18 (0.58)	0.751	0.06 (0.59)	0.914	-0.20 (0.61)	0.739	-0.26 (0.61)	0.670
	Gender		1.80 (2.31)	0.436	1.96 (2.31)	0.394	1.73 (2.28)	0.448	1.87 (2.28)	0.412
	BI				0.35 (0.34)	0.307			0.27 (0.34)	0.429
	Social Anxiety Disorder						1.27 (0.65)	0.053	1.16 (0.67)	0.083
Model fit										
	AIC		2103.39		2963.14		2803.28		3659.69	
	R^2^		0.01		0.02		0.03		0.04	

Note.^a^ In a fifth model, in which BI and Social Anxiety Disordered were entered reversely with Social Anxiety Disorder coming first, results were the same as in Model 4

*The predictor is significant at the *p* <.05 level. ** The predictor is significant at the *p* <.01 level. *** The predictor is significant at the *p* <.001 level

For parent-reported youth anxiety symptoms, BI was a significant predictor of symptoms at post-treatment and 1-year follow-up, with higher levels of anxiety symptoms for those with higher BI scores. Model 2 (age, gender, and BI) seems to have the best fit for predicting post-treatment (AIC = 3059.87, explained variance (*r*^*2*^) = 8.9%) and 1-year follow-up outcomes (AIC = 3006.71, explained variance (*r*^*2*^) = 6.4%). Model 3 (age, gender and social anxiety disorder) seems a better fit at 4-year follow-up (AIC = 2803.28, explained variance (*r*^*2*^) = 3.2%).

The results of the models with CSR as the outcome are shown in Table 4.

**Table 4:** Regression Models Predicting Clinical Severity Ratings at Immediate, 1-Year, and 4-Year Post-Treatment

			Model 1		Model 2		Model 3		Model 4^a^	
			B (SE)	p	B (SE)	p	B (SE)	p	B (SE)	p
			Post-Treatment (*N* = 179)							
Predictors										
	Intercept		3.72 (1.10)	0.001**	1.61 (1.17)	0.167	3.75 (1.08)	0.000	1.83 (1.16)	0.114
	Age		0.08 (0.09)	0.423	0.03 (0.09)	0.770	-0.00 (0.09)	0.984	-0.03 (0.09)	0.775
	Gender		0.25 (0.39)	0.521	0.32 (0.37)	0.395	0.21 (0.38)	0.581	0.28 (0.37)	0.445
	BI				0.22 (0.05)	0.000***			0.19 (0.05)	0.000***
	Social Anxiety Disorder						0.30 (0.11)	0.007**	0.23 (0.11)	0.038*
Model fit										
	AIC		1743.24		2588.72		2439.74		3284.38	
	R^2^		0.01		0.12		0.05		0.14	
			1-Year Follow-Up (*N* = 179)							
Predictors										
	Intercept		1.16 (1.18)	0.326	-0.76 (1.24)	0.541	1.19 (1.13)	0.291	-0.46 (1.21)	0.704
	Age		0.22 (0.10)	0.029*	0.15 (0.09)	0.123	0.11 (0.10)	0.261	0.08 (0.09)	0.441
	Gender		0.02 (0.42)	0.970	0.05 (0.41)	0.903	-0.03 (0.41)	0.945	0.01 (0.39)	0.984
	BI				0.22 (0.06)	0.000***			0.19 (0.06)	0.001**
	Social Anxiety Disorder						0.43 (0.12)	0.000***	0.35 (0.12)	0.002**
Model fit										
	AIC		1728.39		2576.23		2418.93		3266.13	
	R^2^		0.03		0.12		0.12		0.18	
			4-Year Follow-Up (*N* = 179)							
Predictors										
	Intercept		1.51 (1.36)	0.264	0.99 (1.52)	0.512	1.82 (1.30)	0.162	1.62 (1.48)	0.273
	Age		0.05 (0.12)	0.694	0.03 (0.12)	0.793	-0.12 (0.12)	0.339	-0.12 (0.12)	0.327
	Gender		0.53 (0.50)	0.295	0.54 (0.50)	0.281	0.51 (0.48)	0.288	0.52 (0.48)	0.283
	BI				0.06 (0.07)	0.457			0.02 (0.07)	0.767
	Social Anxiety Disorder						0.53 (0.14)	0.000***	0.53 (0.15)	0.000***
Model fit										
	AIC		1853.19		2713.43		2543.64		3400.60	
	R^2^		0.01		0.01		0.09		0.09	

Note.^a^ In a fifth model, in which BI and Social Anxiety Disordered were entered reversely with Social Anxiety Disorder coming first, results were the same as in Model 4. *The predictor is significant at the *p* <.05 level. ** The predictor is significant at the *p* <.01 level. *** The predictor is significant at the *p* <.001 level

Having social anxiety disorder was a significant predictor of CSR at all assessment points, with higher clinical severity for those with social anxiety disorder. Higher BI significantly predicted higher CSR at post-treatment and 1-year follow-up. Model 4 (age, gender, BI, and social anxiety disorder) seems to have the best fit for predicting outcomes at post-treatment (AIC = 3284.38, explained variance (*r*^*2*^) *=* 13.9%) and 1-year follow-up (AIC = 3266.13, explained variance (*r*^*2*^) = 17.7%). Model 3 (age, gender and social anxiety disorder) seems a better fit at 4-year follow-up (AIC = 2543.64, explained variance (*r*^*2*^*)* = 9.2%).

### Predictors of Diagnostic Recovery

The results of the models with diagnostic recovery as outcome are shown in Table 5.

**Table 5:** Logistic Regression Models Predicting Diagnostic Recovery at Post-Treatment, 12-months, and 4-year Follow-Up

		Model 1		Model 2		Model 3		Model 4^a^	
		OR	95% CI	OR	95% CI	OR	95% CI	OR	95% CI
		Post-Treatment (*N* = 179)							
Predictors									
	Intercept	0.54	0.06,4.84	1.44	0.13,16.38	0.48	0.05,4.57	1.11	0.09,13.27
	Age	0.99	0.83,1.18	1.02	0.85,1.21	1.06	0.87,1.28	1.07	0.89,1.29
	Gender	0.81	0.39,1.68	0.79	0.38,1.64	0.83	0.39,1.74	0.82	0.39,1.72
	BI			0.99*	0.98,0.99			0.99	0.98,1.00
	Social Anxiety Disorder					0.78*	0.64,0.96	0.81*	0.65,0.99
Model fit									
	AIC	184.15		182.19		180.52		180.01	
		1-Year Follow-Up (*N* = 179)							
Predictors									
	Intercept	8.13*	1.08,66.62	27.6**	2.81,310.46	8.29*	1.04,71.72	22.19*	2.16,259.16
	Age	0.83*	0.70,0.98	0.85	0.72,1.00	0.89	0.74,1.05	0.89	0.75,1.06
	Gender	0.88	0.45,1.72	0.86	0.43,1.70	0.91	0.46,1.82	0.88	0.44,1.77
	BI			0.99*	0.98,0.99			0.99	0.98,1.00
	Social Anxiety Disorder					0.76**	0.62,0.92	0.79*	0.64,0.97
Model fit									
	AIC	201.61		197.55		195.90		194.26	
		4-Year Follow-Up (*N* = 179)							
Predictors									
	Intercept	4.04	0.64,26.69	6.19	0.81,50.47	3.65	0.54,25.75	4.31	0.52,37.68
	Age	0.93	0.79,1.08	0.94	0.81,1.09	1.03	0.87,1.21	1.03	0.87,1.22
	Gender	0.72	0.38,1.36	0.70	0.37,1.34	0.71	0.36,1.37	0.70	0.36,1.36
	BI			0.99	0.99,1.00			0.99	0.99,1.01
	Social Anxiety Disorder					0.71**	0.57,0.87	0.72**	0.58,0.88
Model fit									
	AIC	223.96		225.01		214.96		216.84	

Note.^a^ In a fifth model, in which BI and Social Anxiety Disordered were entered reversely with Social Anxiety Disorder coming first, results were the same as in Model 4

*The predictor is significant at the *p* <.05-level. ** The predictor is significant at the *p* <.01-level. *** The predictor is significant at the *p* <.001-level

Having social anxiety disorder in the diagnostic profile at baseline predicted a lower probability of diagnostic recovery (from all anxiety disorders) at all assessment points. Higher BI significantly predicted a lower probability of complete recovery at post treatment and 1-year follow-up (model 2), though not when social anxiety was added (model 4). Model 4 (age, gender, BI, and social anxiety disorder) seems to have the best fit for predicting outcomes at post-treatment and 1-year follow-up (AIC = 180.01 and 194.26). Model 3 (age, gender and social anxiety disorder) seems a better fit at 4-year follow-up (AIC = 214.96).

## Discussion

In this study, we investigated if BI and a diagnosis of social anxiety predicted outcomes among youth with anxiety disorders receiving CBT. We found that BI and social anxiety disorder appear to be unique predictors of CBT outcome, differing in relation to both assessment points and outcome measures. Higher BI predicted higher reported symptom levels and clinical severity ratings at post-treatment and 1-year follow-up. Social anxiety disorder predicted higher CSR and negatively predicted diagnostic recovery at all assessment points after treatment. Both BI and social anxiety disorder predicted CSR and diagnostic recovery at post-treatment and 1-year follow-up, and higher BI only significantly negatively predicted diagnostic recovery at post-treatment and 1-year follow-up without social anxiety disorder in the model. Social anxiety disorder did not predict symptom levels at any assessment point, and BI did not predict any outcomes at 4-year follow-up.

Controlling for social anxiety disorder, we found that higher BI predicted higher levels of CSR and higher levels of parent-reported youth anxiety symptoms post-treatment and at 1-year follow-up. In addition, higher BI predicted higher levels of youth-reported youth anxiety symptoms at 1-year follow-up. These results are in line with reports on previous studies of CBT outcomes among preschool children with high BI (Hirshfeldt-Becker et al., 2010; Morgan et al., 2018). Our findings indicate that the effect of therapy on youth with higher BI is either lower or less observable in a short-term perspective. It could also be that among youth with higher BI, some anxiety symptoms and related behaviors are more ego-syntonic, aligned with the youths’ natural way of being in the world, and thus harder to identify and address (Tillfors & Ekselius, 2009).

Youth-reported symptom levels were only predicted by BI at 1-year follow-up. It may be that an immediate experience of symptom reduction lasts shorter among youth with higher BI. Though comparable post-treatment youth reported symptom levels across BI levels, youth with higher BI may be more prone to relapse after one year, while the treatment effect could be more even across BI levels after a longer period.

At 4-year follow-up, BI was not a significant predictor of any outcome measures. This could mean that despite poorer treatment outcomes in a short-term perspective, youth with higher BI levels have treatment outcomes comparable to those of youth with lower BI in the longer term. Youth with higher BI may need more time for treatment to change their habitual way of thinking and behaving. It is also possible that the impact of BI diminishes with age (Balle et al., 2022). These results indicate that youth with a history of higher BI benefit from treatment, which has been documented for youth with high BI and specific phobias and preschool children exposed to prevention interventions (Capriola et al., 2017; Ooi et al., 2022).

Having social anxiety disorder negatively predicted diagnostic recovery and predicted higher CSR at all assessment points. These findings are in line with the relationship between social anxiety and poorer treatment outcomes seen in other studies and raise the questions of why youth with social anxiety disorder do not benefit more from CBT, and how we can improve their treatment (Evans et al., 2021). The specific social anxiety maintaining processes of safety behaviors and self-focused attention are probable explanations for lower treatment outcomes, as they may not be sufficiently addressed in generic CBT programs (Evans et al., 2021). In their cognitive model of social anxiety disorder, Clark and Wells (1995) focused on these specific maintaining factors, as they could hinder the potential learning effect of exposure tasks. Their treatment model has been applied to youth and includes attentional training, behavioral experiments and target persistent negative self-evaluations (Leigh & Clark, 2016; Warnock-Parkes et al., 2022). The cognitive model has shown promising results compared to group CBT and attentional placebo for adolescent social anxiety (Leigh & Clark, 2016; Ingul et al., 2014).

Gradual exposure based on a habituation paradigm may not be sufficient in social anxiety disorder treatment (Arch & Craske, 2008). The use of safety behaviors may prevent learning in exposure tasks and maintain the disorder by establishing dependency on safety strategies to avoid the feared scenario (Craske et al., 2014). According to inhibitory learning models, the individual needs to learn that the feared outcome does not happen despite strong fear to be able to use the new learning in later situations when fear is high. This is more in line with cognitive models that emphasize behavioral testing to disconfirm beliefs and assumptions (Craske et al., 2014).

Another specific social anxiety treatment program is Social Effectiveness Therapy (SET), which has been found superior to exposure therapy alone for social anxiety disorder with recovery rates of 67% compared to 54% (Beidel et al., 2014). In addition to psychoeducation and exposure tasks, SET involves extensive social skills training with rehearsal and feedback and may share some of the mechanisms of behavioral experiments, replacing self-focus and safety behaviors with relational presence and more adaptive social skills.

We found that social anxiety disorder did not predict symptom ratings at any assessment point, while BI did. Thus, there is a different pattern in how the two constructs relate to the specific outcome measures and assessment points. Whereas definitions of temperament have often focused on descriptions of observable behavior in preverbal years, definitions of disorder involve a combination of behavior and subjective experience (Rapee et al., 2005). When assessment of temperament in youth is based on self-report, the distinction between social anxiety disorder and BI is more complex. BI and social anxiety disorder can be separated by the necessity of significant distress or life interference for the assignment of a diagnosis, which is not required for high BI (Rapee et al., 2005). In light of this, the lack of prediction of symptom levels from social anxiety disorder could be related to the degree of functional impairment that anxiety disorders imply. The functional impairment may result in avoidance of anxiety-provoking situations, and resignation towards social situations leading to overshadowing some anxiety symptoms as in comorbid depression (Rotge et al., 2011). Extensive use of avoidance and safety strategies may mask the symptoms or delay the outbreak of anxiety symptoms as well as the potential correctional experience of the feared situation (Evans et al., 2021; Wong & Rapee, 2016). These explanations could be combined with the documented underreporting or denial of symptoms and functional impairment among youth with social anxiety disorder, and the use of diagnostic interviews and clinical experience is therefore important in the assessment of youth anxiety (Aune et al., 2022).

Our finding of a more consistent influence on diagnostic recovery of social anxiety disorder compared to BI and a stronger influence of BI on symptom scores could result from the measurement methods. The clinical evaluation of severity and function in the diagnostic assessment at baseline captures the overall severity, while parents retrospectively reported BI does not, and the latter may be more alike parent reports of youth anxiety symptoms.

According to the current findings on the relation from BI and social anxiety disorder to CBT outcomes, there seems to be a need to adjust CBT for youth with high BI and/or social anxiety disorder. Similar conclusions have previously been drawn separately for BI and social anxiety disorders (Capriola et al., 2017; Evans et al., 2021).

### Strengths and Limitations

The current study addresses a clinically relevant question concerning the relations from BI and social anxiety disorder to CBT outcomes, aiming to better reach youth with anxiety that have modest effects of generic CBT. It has a specific measure of childhood BI, and both anxiety levels and diagnostic outcomes in a 4-year perspective after treatment in an RCT design with a considerable sample from community clinics.

The use of retrospective parent-reported BI may lead to a larger margin of error, as opposed to professional observational rating in infancy or preschool age. However, previous studies have not found effects of age and time on BI- assessment (Clauss & Blackford, 2012; Sandstrom et al., 2020). The use of retrospective parent reports of BI has previously been supported (Reznick et al., 1992). Still, it is uncertain to what degree the BI scores in our study reflect a stable temperamental predisposition in youth, a former temperament that has changed, or a distant memory skewed by the present parental impression of the youth.

Further, the study had limited control over factors affecting the development of symptoms and functioning across the assessment points. The generalizability of the results is limited by the homogenous sample of Norwegian youth with low cultural diversity.

### Clinical Implications

In the current study, BI and social anxiety disorder were found to be negative predictors of treatment outcomes among youth with common anxiety diagnoses. BI seemed to indicate poorer immediate response across outcomes. Clinicians should be made aware of the specific and shared nature of BI and social anxiety disorder that might hinder treatment effects across a broader spectrum of anxiety-related diagnoses. There may be a need to consider adjustments of interventions for differential temperamental groups and to target the specific dynamics of social anxiety better (Capriola et al., 2017; Evans et al., 2021). As BI and social anxiety disorder share features that are proposed as maintaining factors of social anxiety disorder, identifying and targeting specific patterns of avoidance and safety behaviors related to social situations such as self-focused attention and negative observer-perspective images could be crucial for youth with either high BI, social anxiety disorder, or both (Clark & Wells, 1995; Evans et al., 2021; Leigh & Clark, 2018; Spence & Rapee, 2016). Self-focused attention and self-scrutinizing tendencies are similar to the proposed factors of risk and resilience toward later anxiety among children with BI, and their (potential) amendment via CBT seems relevant to therapeutic outcomes in this group (Fox et al., 2023).

As BI is an innate temperamental trait that predisposes for avoidance of a range of situations from preverbal years, maintaining patterns of safety strategies may be particularly hard to identify and change (Tillfors & Ekselius, 2009). Youth with high BI may need more time to achieve a better understanding of their safety strategies and attentional foci, how these affects them, and how to recognize differential versions of these patterns in their everyday life. Youth with high BI may also need sufficient differential and repeated practical experience with alternative strategies, attentional foci and social skills, focusing on implementation and maintenance of these in differential situations in the future (Beidel et al., 2014; Leigh & Clark, 2018; Warnock-Parkes et al., 2022). Whether youth with high BI and/or social anxiety disorder need additional time or contents *in* treatment, additional time or skills to incorporate treatment achievements *after* treatment, or both, is uncertain.

We suggest clinicians consider the following elements when treating youth with anxiety disorders: (1) recognizing and targeting specific features of BI and social anxiety, (2) striving towards sufficient time for youth to identify a broad range of their specific maintaining factors across different settings, (3) striving towards sufficient time and repetition of experiences that challenge the maintaining strategies in a broad range of situations, and (4) expanding and specifying the work on relapse prevention by establishing routines that help youth continue to identify tendencies of avoidance and experiment with their attentional focus and social skills in a broad range of situations after treatment is ended. These may be steps toward developing a more specific and comprehensive approach to target the mechanisms behind social withdrawal, self-criticism, and avoidance across differential groups with clinical anxiety.

## Conclusion

In addition to social anxiety disorder, higher BI appears to represent a risk for poorer outcomes of CBT for youth anxiety. Our findings indicate two slightly different outcome profiles for BI and social anxiety disorder. Having social anxiety seems associated with a lower probability of recovery both in the short and long term. Having higher levels of BI seems to indicate that an immediate effect on symptom levels is delayed or will vanish quite fast and that a longer time is needed for changes in symptom levels to be established. The combination of having high BI and social anxiety disorder may indicate the need for treatment adjustments. Further studies are needed to examine the differential influence of BI and social anxiety disorder on treatment outcomes over time and explore CBT adjustments for youth with anxiety disorders.
